# Influence of artificially induced light pollution on the hormone system of two common fish species, perch and roach, in a rural habitat

**DOI:** 10.1093/conphys/coy016

**Published:** 2018-04-13

**Authors:** Anika Brüning, Werner Kloas, Torsten Preuer, Franz Hölker

**Affiliations:** 1 Leibniz-Institute of Freshwater Ecology and Inland Fisheries, Müggelseedamm 310, 12587 Berlin, Germany; 2 German Federal Institute for Risk Assessment, Max-Dohrn-Str. 8-10, 10589 Berlin, Germany

**Keywords:** Fish, gonadotropins, light pollution, melatonin, sex steroids

## Abstract

Almost all life on earth has adapted to natural cycles of light and dark by evolving circadian and circannual rhythms to synchronize behavioural and physiological processes with the environment. Artificial light at night (ALAN) is suspected to interfere with these rhythms. In this study we examined the influence of ALAN on nocturnal melatonin and sex steroid blood concentrations and mRNA expression of gonadotropins in the pituitary of European perch (*Perca fluviatilis*) and roach (*Rutilus rutilus*). In a rural experimental setting, fish were held in net cages in drainage channels experiencing either additional ALAN of ~15 lx at the water surface or natural light conditions at half-moon. No differences in melatonin concentrations between ALAN and natural conditions were detected. However, blood concentration of sex steroids (17β-estradiol; 11-ketotestosterone) as well as mRNA expression of gonadotropins (luteinizing hormone, follicle stimulating hormone) was reduced in both fish species. We conclude that ALAN can disturb biological rhythms in fish in urban waters. However, impacts on melatonin rhythm might have been blurred by individual differences, sampling methods and moonlight. The effect of ALAN on biomarkers of reproduction suggests a photo-labile period around the onset of gonadogenesis, including the experimental period (August). Light pollution therefore has a great potential to influence crucial life history traits with unpredictable outcome for fish population dynamics.

## Teaser

Natural light is an important trigger for circadian and circannual rhythms, which can be disturbed by light pollution. Low intensity artificial light at night suppresses different hormones of the reproductive axis of two common freshwater fish species, the European perch, *Perca fluviatilis*, and roach, *Rutilus rutilus*, in a natural setting.

## Introduction

Light is fundamental for the existence of flora and fauna on earth. It serves not only as a source of energy but also as a source of information to organisms that drives daily and seasonal cycles of behaviour, phenology and physiological change. The natural variation in the length of the day and night sets the internal clock of organisms and ensures that behaviours and physiological processes are synchronized with the time of day and the season (Gaston *et al.*, 2013). This is particularly important in reproduction of many animals, for example, in timing of courtship and mating, which ensures that rearing of offspring can be coordinated with the availability of natural resources, optimizing survival.

However, especially in urban areas, nightscapes are increasingly polluted by artificial light at night (ALAN) (Hölker *et al.*, 2010a; Kyba *et al.*, 2017a). Streetlights, illuminated advertising and other kinds of public lighting are causing direct glare. Furthermore, since they are frequently unshielded against the sky, their irradiance is reflected by airborne particles, aerosols and other molecules. The result is that the nocturnal urban sky can be brighter than a full moon night (Kyba *et al.*, 2011, 2015). Ecological consequences are known for almost all classes of organisms (Rich and Longcore, 2006; Schroer and Hölker, 2016). Impacts on humans, such as psychological, cardiovascular and metabolic alterations or even an increased cancer risk, have also been discussed (Haim and Portnov, 2013; Cho *et al.*, 2015). Birds and insects that migrate at night can be greatly attracted to artificial light, become trapped and lose important energy reserves (Eisenbeis, 2006; Gauthreaux and Belser, 2006; La Sorte *et al.*, 2017). Also impacts on reproductive physiology and behaviour have been reported (Botha *et al.*, 2017; McLay *et al.*, 2017).

In aquatic environments, impacts of ALAN on the biomass and composition of primary producers in benthic communities have been found (Grubisic *et al.*, 2017). Also microbial diversity and respiration are altered under the influence of ALAN (Hölker *et al.*, 2015). Zooplankton, such as daphnids, exhibit changes in their daily vertical migration pattern in response to ALAN at the very low light levels produced by skyglow (Moore *et al.*, 2006). Drift patterns of aquatic insect larvae are likewise modified by ALAN at ~1 lx (Perkin *et al.*, 2014b). In sum, this may lead to general changes of food web interactions and ecosystems functions (Perkin *et al.*, 2011).

In fish, many behavioural and physiological activities underlie either daily or seasonal rhythms. The European eel (*Anguilla anguilla*), for example, performs a spawning migration from freshwater to the ocean between autumn and spring. Eels migrate during dark nights and rest during bright nights around full moon (Stein *et al.*, 2015). ALAN is able to disrupt this migration (Lowe, 1952; Vøllestad *et al.*, 1986), which is also known for other migratory fish species such as salmon (*Salmo salar*) (Hansen and Jonsson, 1985; Greenstreet, 1992). Thus, it is reasonable to assume that illuminated bridges or embankment lighting can interfere with traits relevant to reproduction. In fish, several reproductive traits are controlled by internal clocks and depend on light as an important cue. Along with other photoreceptor systems and endogenous oscillators, the photosensitive pineal complex is considered to be highly important in regulating rhythmicity (Ekström and Meissl, 1997; Falcón *et al.*, 2010). The daily fluctuations of the ‘night’ hormone melatonin always follow the photoperiodical changes. Since melatonin secretion is suppressed by light, levels are high at night and low during daytime. Thus, the pineal complex transduces photoperiodic information into hormonal signals (here melatonin) that can be used by other organs to synchronize physiological and behavioural processes with daytime and season.

It is commonly accepted that light plays a key role in mediating reproductive processes in temperate freshwater fish. It is a common practice in science and aquaculture to control these processes by manipulating the photoperiod with artificial light. Consequently, research is focusing on the interaction between light and reproduction (Kolkovski and Dabrowski, 1998; Porter *et al.*, 1998, 1999; Zakes and Szczepkowski, 2004; Maitra *et al.*, 2013). Gametogenesis and final maturation are regulated by a hormonal cascade of the brain–pituitary–gonad (BPG) axis, including gonadotropin-releasing hormone (GnRH), pituitary gonadotropins, luteinizing hormone (LH), follicle stimulating hormone (FSH) and sex steroids. Light is known to manipulate these axis components. In general, maturation, onset of gonadogenesis, or spawning events can be controlled by photoperiod manipulation. Fish farmers and scientists are using this to induce off-season spawning in fish that normally reproduce only once per year or to prevent precocity in commercial aquaculture species (Carrillo *et al.*, 2009; Kolkovski and Dabrowski, 1998; Macquarrie *et al.*, 1979; Rodríguez *et al.*, 2005).

However, this kind of research has predominantly been conducted in the lab or in artificial environments with high intensity night lighting. The potential impacts of ALAN on fish in a natural context at typical light levels found in light-polluted areas are not well studied. In previous experiments we assessed the impact of low light intensity ALAN on the circadian rhythm of melatonin and mRNA expression of gonadotropins of European perch (Brüning *et al.*, 2015, 2016) and roach (Brüning *et al.*, 2018) under laboratory conditions. We found that the melatonin rhythm was substantially diminished even at light intensities as low as 1 lx. Furthermore, mRNA expression of LH and FSH was significantly impaired in perch.

Falcón *et al.* (2010) reviewed the possible interactions between melatonin and the BPG axis and suggested that fishes possess a photo-labile period. One such period in fish species of temperate waters is autumn, when daylength is decreasing. In this period, Falcón *et al.* suggest fish are susceptible to photoperiod manipulation, and continuous light decreases synthesis and secretion of gonadotropins and sex steroids, causing gonadal maturation to fail.

In the present field study ALAN is represented by streetlights along an embankment in a naturally dark environment.

The experimental fish species are European perch and roach, two of the most abundant fish species in European freshwaters, which inhabit a wide range of habitats, including all types of lakes and most streams and ditches (Kottelat and Freyhof, 2007).

European perch are known to feed on zooplankton when young, but undergo an ontogenetic niche shift towards a diet of benthic organisms and fish when older (Persson, 1986). They are diurnal and crepuscular consumers. During the daytime perch can be found in pelagic and littoral zones of lakes whereas at night high numbers of motionless perch are often observed at the bottom of shallow littoral areas where ALAN could become a relevant stressor (Hölker *et al.*, 2007). Gonadogenesis begins in late summer, around the end of August and September (Sulistyo *et al.*, 1998, 2000) and spawning takes place during April and May (Treasurer, 1988; Wang and Eckmann, 1994).

Roach are also diurnal and crepuscular (Hölker and Breckling, 2005), but nocturnal feeding has been reported for roach in periurban lakes experiencing ALAN (Okun and Mehner, 2005). They feed on benthic invertebrates, zooplankton and plant material. Roach often undertake diel horizontal migrations between the relatively safe and shallow littoral lake habitats during the daytime towards the more risky, but also more profitable upper layer of the pelagic zone at night (Hölker *et al.*, 2007). Gonadogenesis appears to begin around August and spawning takes place between April and May (Trubiroha *et al.*, 2012).

Because many ditches and small streams are quite shallow, both perch and roach could be particularly vulnerable to exposure to artificial light throughout the year. The objective of this study was to investigate the effect of ALAN on perch and roach in a natural context, especially on melatonin and reproductive hormones during a suspected photo-labile period in late summer (August/September). We hypothesize that ALAN experienced during this period is able to significantly decrease levels of melatonin and sex steroids [11-ketotestosterone (11-KT) and 17β-estradiol (E2)] in the blood and significantly reduce mRNA expression of gonadotropins (LH, FSH) in the pituitaries of the experimental fish.

## Methods

### Field site

The field site is situated ~70 km north-west of Berlin, Germany, in the Westhavelland Nature Park (52′69° N, 12′46° E). The Nature Park is one of the least-illuminated areas of Germany and has recently been designated an ‘International Dark-Sky Reserve’ by the International Dark-Sky Association (IDA) (www.darksky.org).

The experimental field sites consisted of two light-naïve grassland fields along a drainage ditch that were under agricultural use (mown twice per year, not fertilized). The ditch itself is ~5 m wide and 50–80 cm deep, depending on precipitation. Each of the fields was equipped with 12 streetlights (20 m apart) in 3 rows parallel to the ditch. The first row (four streetlights) was installed 3 m away from the edge of the water. On one field (lit field) the streetlights were equipped with high-pressure sodium lamps at 4.75 m height (70 W, 2000 K, 96 l mW^−1^; Osram Vialox NAV-T Super 4Y, Munich, Germany; Holzhauer *et al.*, 2015). The spectrum of the lamps can be found in Fig. 3 in Holzhauer *et al.* (2015). The high-pressure sodium lamps show peaks in the blue-green, green, yellow and red parts of the visible spectrum.

Nocturnal light levels in the lit field ranged from 13.3 to 16.5 lx at the water surface and 6.8–8.5 lx at 50 cm depth (Hölker *et al.*, 2015). The control field experienced natural light levels between ~0.002 lx (new moon) and 0.1 lx (full moon) at the water surface and between 0.001 and 0.05 lx at 50 cm depth, respectively. The experimental sites are separated by 600 m (Euclidian distance) and a row of trees ensures that there is no influence of the experimental lights on the control site (Hölker *et al.*, 2015) The streetlights in the other field (dark field) were not equipped with lamps.

In front of each streetlight at the first row along the ditch, two net cages (1 × 1 × 1 m^3^, aluminium frame with nylon net, 1 mm mesh size) were installed in the ditch, ~1 m away from the water edge, thus in total there were eight net cages per field. Water depth during the experimental period was ~80 cm, so that the cages covered the water column from bottom to surface. The two sites were similar in terms of morphology, catchment characteristics and abiotic parameters (Holzhauer *et al.*, 2015). On 7 August 2012 each net cage was equipped with six perch and six roach for 1 month.

### Experimental fish

European perch, *Perca fluviatilis*, and roach, *Rutilus rutilus*, from the stock of the Leibniz Institute of Freshwater Ecology and Inland Fisheries (IGB) in Berlin, Germany, originated from the nearby Lake Müggelsee, a periurban lake surrounded by forests and housing and thus partly experiencing ALAN (Perkin *et al.*, 2014a). Before transportation to the field site fish were kept in indoor tanks for ~4 weeks (perch) and several months (roach) in a flow-through system with tap water and aeration under natural photoperiod (natural light through windows). They were fed daily with frozen chironomid larvae (perch) and dry food (roach). Temperature was 15.5°C and water parameters were regularly controlled to maintain optimum conditions. The mean fish biomass was 32.1 ± 13.2 g (mean ± standard deviation) for perch and 29.6 ± 11.9 g for roach.

### Blood and tissue sampling

Sampling took place in 4 consecutive nights around half-moon from 6–9 September 2012. Due to the half-moon, natural nocturnal light levels of up to 0.02 lx were measured at the water surface of the control site. Four cages per night were sampled, two from each field in randomized order. Sampling took place between 21:00 in the evening and 05:00 in the morning.

The fish were taken out with a landing net and transferred to a black bucket with ice water covered with a light-proof lid. Sampling was carried out with dim white LED-light switched on only shortly for orientation and to check if cages were empty. Fish were immediately sampled in a transporter equipped with a mobile laboratory.

Blood samples were taken with heparinized syringes from the caudal vein and centrifuged at 7000 rpm for 2 min. The serum was stored in a light-proof polystyrene box containing a freezing mixture (crushed ice + NaCl, −20°C) for the duration of the sampling and transferred to a freezer thereafter. Fish were weighed and measured. After decapitation, the pituitary was dissected and transferred to RNAlater (Sigma-Aldrich) to preserve the mRNA. The pituitary tissue was left in RNAlater for ~24 h at 4°C and frozen thereafter. The sex of the fish was determined by inspection of gonads. All vessels containing blood and tissue samples were stored and transported in light-proof cryoboxes.

### Hormone extraction and assay

Blood serum samples (70–100 μl) were transferred to 5ml glass vials and 500 μl ethyl acetate (J.T.Baker) was added to each vial. Each vial was vortexed for 30 s and then left for 5 min to allow phase separation. Afterwards the vials were frozen at −80°C for 15 min and the liquid phase was transferred to a new vial. The procedure was repeated once and the supernatant of both extractions was pooled for each sample.

The supernatant was then evaporated under a stream of nitrogen at 45°C. The residue was reconstituted with 0.5 mL EIA-buffer (1 M phosphate solution containing 1% BSA, 4 M sodium chloride, 10 mM EDTA and 0.1% sodium azide, Cayman Chemicals).

Melatonin, E2 and 11-KT levels were measured by enzyme linked immunosorbent assay (ELISA) using commercial kits [RE54021, RE52041, CM582751 (Cayman Chemicals), IBL, Hamburg, Germany].

### RNA extraction and reverse transcription

RNA extraction and reverse transcription followed the protocol used in Brüning *et al.* (2015). Total pituitary RNA was extracted with RNeasy extraction kit (Qiagen) following the manufacturers protocol. Concentration of total RNA was measured by UV absorption spectrometry with a Nanodrop ND-1000 spectrophotometer (Thermo Fisher Scientific).

Reverse transcription of total RNA was performed using Affinity Script Multiple Temperature Reverse Transcriptase (Stratagene).

### mRNA expression analysis by RT-qPCR and relative mRNA quantification

RT-qPCR and relative mRNA quantification followed the protocol used in Brüning *et al.* (2015). For LHß, FSHβ and ribosomal protein L8 (rpL8) as housekeeping gene, we used primers established by Trubiroha *et al.* (2009) for roach and for perch we used primers established by Brüning *et al.* (2016) (Table 1). The identity of the products was confirmed by direct sequencing (SEQULAB, Göttingen) and comparison with the database homology search tool BLAST.

**Table 1:** Overview of primer specific PCR conditions (primer sequences L8, FSHß, LHß)

	Target gene	Forward primer	Reverse primer	*T_A_* (°C)	Primer conc. (nM)	Product size (bp)	PCR-efficiency
*Perca fluviatilis*	L8	GTTATCGCCTCTGCCAAC	ACCGAAGGGATGCTCAAC	62	375	167	2.02
	FSH	CCTACTGGCAGGGAAGAAC	CCTACTGGCAGGGAAGAAC	64	375	85	1.92
	LH	GGCTGTCCAAAGTGTCACCT	GGGAGAACAGTCAGGGAGCTTAA	62	188	158	1.9
*Rutilus rutilus*	L8	ATCCCGAGACCAAGAAATCCAGAG	CCAGCAACAACACCAACAACAG	62	375	94	1.98
	FSH	CTGTCGGCTTTCCAATATC	GGCTACGGTAAACTCTTTC	62	375	119	1.93
	LH	TAGGTGATGTGCGGGTCCAC	AAGAGCTGTCCGAAATGC	62	375	187	1.94

*T_A_*—annealing temperature.

PCR was carried as previously described by Brüning *et al.* (2016). PCR efficiencies were determined with pooled pituitary cDNA and ranged between 1.90 and 2.02; mRNA expression was determined by comparative CT method (ΔΔCT) with using a calibrator sample (pooled pituitary cDNA) and correction for the PCR efficiency according to Pfaffl (2001).

### Data handling and statistical analysis

Hormone data were standardized to pg ml^−1^. After log-transformation of the hormone data to meet the assumptions of the test (normality of residuals), hormone and mRNA expression data were analysed using Linear Mixed Models (LMM, fixed factor: treatment; random factor: cage number, to account for possible variation between and dependency of fish within cages). The statistical tests were performed with IBM SPSS Statistics (Version 19).

## Results

### Melatonin

**Figure 1:**
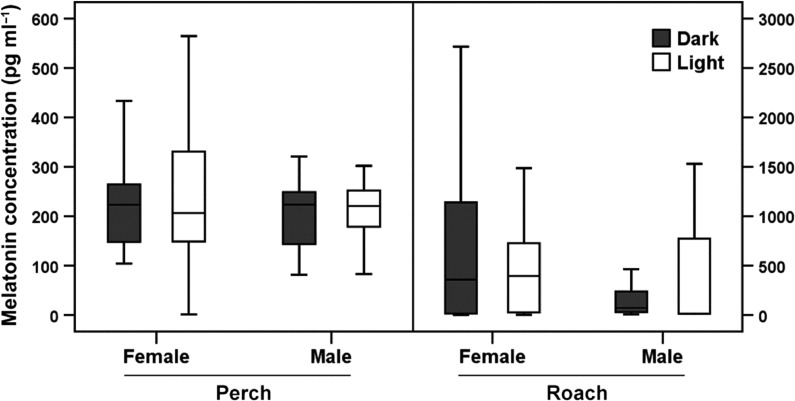
Concentrations of melatonin in blood serum. Comparison between effects of ALAN (‘Light’) and natural half-moon conditions (‘Dark’) on female [light: *N* (number of fish) = 22; dark: *N* = 23] and male (light: *N* = 5; dark: *N* = 19) perch and female (light: *N* = 33; dark: *N* = 35) and male (light: *N* = 3; dark: *N* = 5) roach. Data is presented as box plots [Box: median, IQR (interquartile range); whisker: 5–95% values]. No significant differences were found.

There were no significant differences in the concentration of melatonin in blood serum of perch subjected to either ALAN or natural half-moon conditions (treatment effect; female: *F*_1,45_ = 0.349, *P* = 0.557; male: *F*_1,24_ = 0.081, *P* = 0.778). Also for roach no significant differences in serum concentrations of melatonin were found (treatment effect; female: *F*_1,13.9_ = 4.469, *P* = 0.053; male: *F*_1,8_ = 0.000, *P* = 0.993) (Fig. 1).

### mRNA expression of gonadotropins

**Figure 2:**
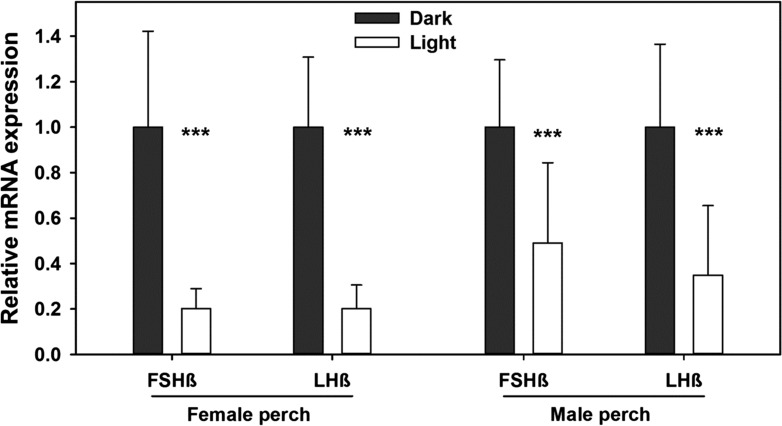
Relative mRNA expression of follicle-stimulating hormone (β-subunit; FSHβ) and luteinizing hormone (β-subunit; LHβ) in brain–pituitary tissue of *P. fluviatilis*, subjected ALAN (‘Light’) or natural half-moon conditions (‘Dark’): female, (dark: *N* = 13, light: *N* = 15) and male (dark: *N* = 13, light: *N* = 8). Data is shown as mean ± SD. Significant differences are represented by asterisks (***P ≤ 0.001). mRNA expression of FSHβ and LHβ was significantly reduced in the ALAN treatment.

**Figure 3:**
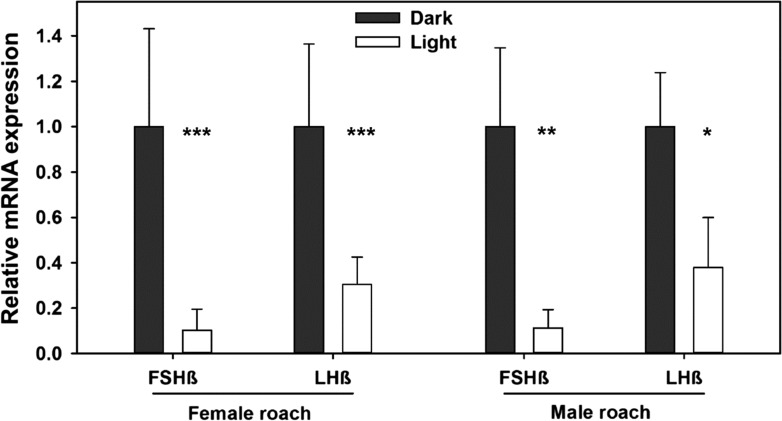
Relative mRNA expression of follicle-stimulating hormone (β-subunit; FSHβ) and luteinizing hormone (β-subunit; LHβ) in brain–pituitary tissue of *R. rutilus* subjected ALAN (‘Light’) or natural half-moon conditions (‘Dark’): female (dark: *N* = 25, light: *N* = 28) and male (dark: *N* = 5, light: *N* = 3) Data is shown as mean ± SD. Significant differences are represented by asterisks (***P ≤ 0.001; **P ≤ 0.01; * *P* ≤ 0.05). mRNA expression of gonadotropins was significantly reduced in the light treatment.

mRNA expression analyses of LHβ and FSHβ from female and male perch subjected to ALAN or no ALAN revealed significant differences in both, FSHß and LHß mRNA expression (FSH: *F*_1,28_ = 55.410, *P* ≤ 0.001 (female) and *F*_1,21_ = 13.923, *P* = 0.001 (male); LH: *F*_1,28_ = 96.980/*F*_1,21_ = 20.063, *P* ≤ 0.001 (male/female)) being highest in the control (dark) treatment and significantly lowered in the ALAN treatment (Fig. 2). Also in roach significant differences in mRNA expression of gonadotropins were found. mRNA expression of FSHß was significantly lowered in the ALAN treatment (female: *F*_1,7.107_ = 26.895, *P* = 0.001; male: *F*_1,6_ = 17.818, *P* = 0.006; Fig. 3). Also LHß expression was significantly lowered by ALAN in female (*F*_1,51_ = 91.274, *P* ≤ 0.001) and male (*F*_1,6_ = 13.260, *P* = 0.011) roach.

### 11-Ketotestosterone

**Figure 4:**
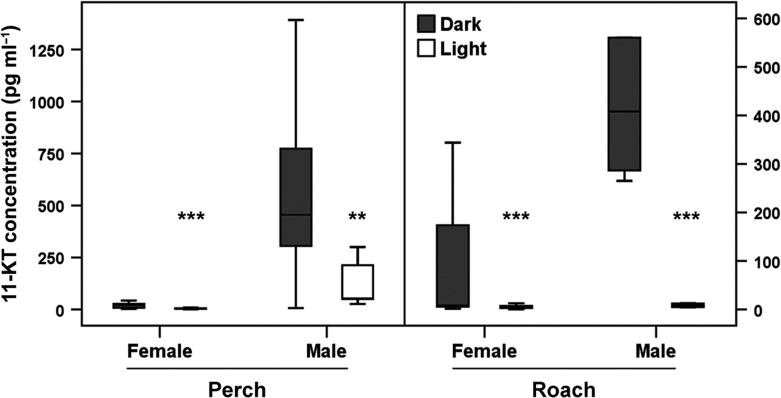
Concentration of 11-KT in blood serum of perch and roach. Comparison between effects of ALAN (‘Light’) and natural half-moon conditions (‘Dark’) on female (light: *N* = 22; dark: *N* = 23) and male (light: *N* = 5; dark: *N* = 19) perch and female (light: *N* = 33; dark: *N* = 35) and male (light: *N* = 3; dark: *N* = 5) roach. Data is presented as box plots [Box: median, IQR (interquartile range); whisker: 5–95% values]. Significant differences are represented by asterisks (***P ≤ 0.001; **P ≤ 0.01). 11-KT concentrations of female and male of both species are significantly reduced in the ALAN-treatment.

The analyses of the blood serum of perch revealed significant differences in 11-KT concentrations between ALAN and control treatment in female (*F*_1,47_ = 12.321, *P* = 0.001) and male (*F*_1,25_ = 8.318, *P* = 0.008). The serum of perch subjected to natural half-moon conditions (control) contained up to 10 times more 11-KT compared to the ALAN treatment (Fig. 4). Similar results were obtained for roach (Fig. 4). 11-KT concentrations in blood serum were likewise significantly lower in the ALAN treatment compared to the control treatment in female (*F*_1,73_ = 15.578, *P* ≤ 0.001) and male roach (*F*_1,9_ = 142.748, *P* ≤ 0.001).

### 17ß-Estradiol

**Figure 5:**
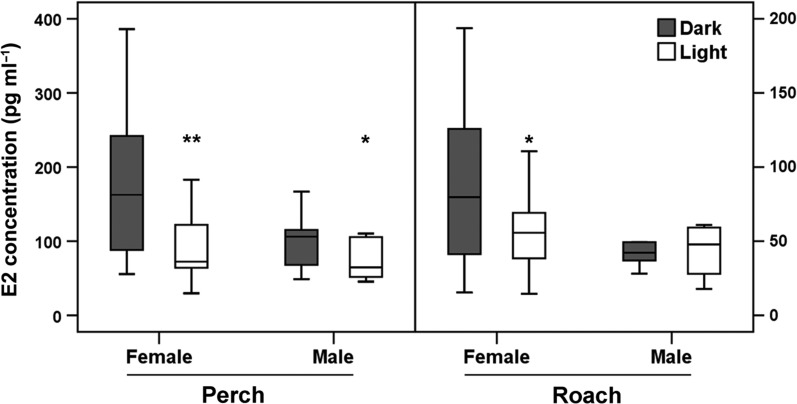
Concentration of E2 in blood serum of perch and roach. Comparison between effects of ALAN (‘Light’) and natural half-moon conditions (‘Dark’) on female (light: *N* = 22; dark: *N* = 23) and male (light: *N* = 5; dark: *N* = 19) perch and female (light: *N* = 33; dark: *N* = 35) and male (light: *N* = 3; dark: *N* = 5) roach. Data is presented as box plots [Box: median, IQR (interquartile range); whisker: 5–95% values]. Significant differences are represented by asterisks (***P ≤ 0.001; **P ≤ 0.01). E2 concentrations are significantly reduced by ALAN in female and male perch and female but not male roach.

The concentrations of E2 were significantly lowered by ALAN compared to the control treatment in female of both, perch (*F*_1,47_ = 7.244, *P* = 0.010) and roach (*F*_1,73_ = 5.981, *P* = 0.017) and in male perch (*F*_1,12.995_ = 7.581, *P* = 0.016). However, E2 concentrations in male roach were not affected by ALAN (*F*_1,9_ = 7.581, *P* = 0.717) (Fig. 5).

## Discussion

This study demonstrated that ALAN had a strong effect on reproductive traits in European perch and roach under *in situ* conditions. mRNA expression of LH and FSH and the production of sex steroids were profoundly affected by ALAN in both species. No effect on serum melatonin was detectable, which seems to contradict our previous results in the lab (Brüning *et al.*, 2015; Brüning *et al.*, 2018) where 1 lx white light already caused a substantial drop in melatonin. However, a NOEC (no observed effect concentration) was not found in the above mentioned study that investigated four different light levels (0, 1, 10 and 100 lx). This suggests that the threshold is lower than 1 lx and even the light emitted at intermediate moon phases may suppress melatonin production. The sampling for the present experiment took place around half moon, when light levels were up to 0.02 lx at the water surface. Although this might initially seem very low, several fish species are known to be affected by very low light intensities. European perch, for example, are still able to capture prey at 0.02 lx (Bergman, 1988). The European eel, *A. anguilla*, in its glass eel stage avoids light levels of 0.07 lx or less (Bardonnet *et al.*, 2005) and the common bream, *Abramis brama*, is able to detect prey at light levels of 0.005 lx (Townsend and Risebrow, 1982). In tench, *Tinca tinca*, melatonin levels dropped to daytime levels after a 1h light pulse of 0.3 lx in the middle of the night (Vera *et al.*, 2005), which is comparable to a full moon scenario (Kyba *et al.*, 2017b). Lunar spawners like rabbitfish, which synchronize their reproduction with the moon, are special cases. The pineal organs of the golden rabbitfish, *Siganus guttatus*, are able to perceive light intensities as low as 0.1 lx (Takemura *et al.*, 2006). Also in the latter two studies, no NOEC was found, suggesting that the threshold light intensities are even lower than the investigated light levels. Perch and roach were observed to use the whole water column during the day and in the light field also during the night. Since the water in the ditches was very shallow and clear during the experiment, it is likely that both perch and roach were able to perceive the natural half-moon intensities via the pineal complex, retinal cells or other melatonin influencing photoreceptors. Thus, melatonin levels in perch and roach might have been influenced by natural half-moon light conditions during sampling. The differences in melatonin levels between ALAN and natural half-moon conditions are therefore smaller than those in the lab experiments in Brüning, *et al.* (2015) where the controls were almost completely dark. The sampling procedure could be another factor responsible for the unexpected results. The sampling of and the movement between the cages (dark field and lit field) took 1.5 h per cage. Consequently, it was not possible to sample each cage at the same time. Together with the high individual variability (Fig. 1), this may have blurred possible differences between the treatments. However, this interpretation remains hypothetical, since empirical data on blood levels of melatonin at different times of day and night and different moon phases are lacking.

Even though the melatonin rhythm of fishes is very susceptible to diurnal and also sudden light changes (Vera, *et al.*, 2005; Vuilleumier *et al.*, 2006), reproduction in fishes from temperate waters is determined by a seasonal rhythm, which is expressed not only by the melatonin rhythm, but also by other hormones and sites such as the thyroid stimulating hormone (TSH), which is rhythmically produced in the saccus vasculosus (Nakane *et al.*, 2013). This might explain why ALAN clearly affected reproductive rhythms in our study (Figs 2–5). Gonadotropins, and thereby sex steroids, are sensitive endpoints when studying reproductive rhythms. Thus, the impact of ALAN on sex steroids and gonadotropins corresponds to our expectations that light pollution in a natural context has a great potential to disrupt crucial rhythms like reproduction in fish.

For the seasonal reproduction rhythm, photoperiod and temperature are regarded as the most important zeitgeber for the majority of species (de Vlaming, 1972; Migaud *et al.*, 2010; Hermelink *et al.*, 2011). Thus, light likely interferes with the BPG axis. In European perch, for example, the decreasing photoperiod in autumn is necessary to induce gametogenesis (Migaud *et al.*, 2003) and an artificially increasing photoperiod or continuous lighting can severely disturb the schedule of reproduction (Fontaine *et al.*, 2006). Studies in salmonids and a Neotropical silverside species have shown that light can act directly on the upper part of the axis, the GnRH by manipulating its timing (Amano *et al.*, 1994; Miranda *et al.*, 2009). The main task of GnRH is to stimulate the expression and secretion of gonadotropins, FSH and LH. Consequently, gonadotropins can be a target for photo-induced disruption too. In the present study the mRNA expression of gonadotropins of European perch and roach was substantially reduced (Figs 2 and 3). In an earlier lab study we already demonstrated that low light intensities suppress gonadotropin expression in perch (Brüning, *et al.*, 2016). As reported in different fish species, gonadotropins play a critical role in the early stages of gonadal maturation, in the synthesis of sex steroids, and consequently in spermatogenesis and vitellogenesis (Patiño and Sullivan, 2002; Schulz and Miura, 2002; Mateos *et al.*, 2003; Yaron *et al.*, 2003). Little data is available on the influence of low intensity ALAN, as referred to as light pollution, on gonadotropin expression in fish. However, gonadotropin mRNA expression was inhibited by continuous light of 650 lx (Rodríguez, *et al.*, 2005), and daily plasma concentrations of LH and its storage were significantly altered by artificial light in European sea bass (*D. labrax*) (Bayarri *et al.*, 2004). These studies used high light intensities as practiced in aquaculture. However, previous studies on perch and roach substantiated the lack of dose response relationship for hormones above 1 lx since little or no additional impact was seen at higher light intensities (10 and 100 lx) (Brüning, *et al.*, 2018). In Brüning *et al.* (2016) we verified a significant reduction in mRNA expression of both gonadotropins at low intensity white (1 lx) and coloured light (0.15–2.2 lx) in perch (*P. fluviatilis*) under artificial conditions. There is also strong evidence that photoperiod manipulation, such as mimicking short or long day photoperiods, can change the timing of gonadotropin production, subsequent spawning events (Breton *et al.*, 1977; Bromage *et al.*, 1982; Felip *et al.*, 2008; Miranda, *et al.*, 2009), and even parental care behaviour (Foster *et al.*, 2016). However, most of these studies were carried out under artificial conditions. The present study is—to our knowledge—the first study, which demonstrates that light pollution in a realistic natural context can suppress gonadotropin mRNA expression in the preparatory phase of reproduction and confirms the previous results of our lab experiments (Brüning, *et al.*, 2016).

One of the major roles of gonadotropins is to trigger the synthesis of sex steroids from the gonads. Sex steroids in turn exhibit positive or negative feedback on gonadotropins and the brain. They are also involved in vitellogenesis, final gamete maturation, and the control of sexual behaviour and spawning (Zohar *et al.*, 2010). In our study the plasma concentrations of 11-KT were significantly reduced by ALAN in both sexes of perch and roach (Fig. 4). Plasma levels of E2 were likewise reduced in the light treatment in female perch, female roach, and male perch (Fig. 5). However, in male roach E2 levels were already low in the control treatment, thus no effect of ALAN was detectable. These findings are in accordance with studies in roach (Trubiroha *et al.*, 2010) where E2 levels in males were significantly lower than in females. The suppression of sex steroids by ALAN has also been reported for other fish species. As shown in male sea bass, continuous light was very effective in inhibiting the increase of 11-KT in plasma during the reproductive cycle (Rodríguez, *et al.*, 2005; Felip, *et al.*, 2008). Also in Senegalese sole plasma E2 levels in female and 11-KT levels in male fish were significantly lower compared to conspecifics under a natural photoperiod (García-López *et al.*, 2006). A concomitant failure in gonad maturation was reported for several fish species, including sea bass (Begtashi *et al.*, 2004), Atlantic cod (Taranger *et al.*, 2006), turbot (Imsland *et al.*, 2003) or Nile tilapia (Rad *et al.*, 2006). Furthermore Migaud *et al.* (2004) found a significant suppression of E2 by high intensity continuous light (500 lx) in male and female perch. These results were obtained in the lab in September (experimental period 1 September—6 October) whereas in July/August (experimental period 17 July—1 September) no differences were found. This is in contrast to our field results where an exposure in August/September (7 August to 6–9 September) elicited a significant suppression. However, Migaud *et al.* (2004) found no differences in plasma 11-KT in either of the two experimental periods. This could be explained by a photo-labile period when fish are particularly susceptible to additional light at night or photoperiod manipulations. Recently, evidence for such a period was found in some fish species. Falcón *et al.* (2010) suggested a photo-labile period for fish in general, coinciding with a species-specific onset of gonadogenesis when photoperiods are increasing or decreasing in spring or autumn. Indeed, in male sea bass this period spans a narrow time frame of ~2 months, including September (Rodríguez *et al.*, 2012). In Atlantic cod photoperiod manipulations were effective when the photoperiod was decreasing in autumn (Davie *et al.*, 2007). In our lab study with roach (winter light conditions), we failed to demonstrate a suppression of gonadotropin expression. In contrast, the experiments with perch under late summer light conditions (onset of gonadogenesis) resulted in a significant suppression of gonadotropin expression with ALAN. The clear inhibition of parts of the BPG axis in perch and roach in this study suggest for both species that the photo-labile period must be the period around the onset of gonadogenesis, including August, when our experiments took place.

Altogether it becomes clear that ALAN can interfere with various components of the BPG axis. Although photoperiod manipulation can be a powerful aquacultural tool for controlling reproductive events, ALAN might be an unpredictable threat for light sensitive species, communities and consequently biodiversity (Hölker *et al.*, 2010b).

Given the fact that the study was performed at only one sampling site, and that other interfering biotic and abiotic factors cannot be completely excluded, the outcome initially applies only for this location. We tried to capture potential effects by carefully monitoring important environmental variables in the two experimental fields (Holzhauer *et al.* 2015). Since we found no differences likely to influence the experiments, we can confidently ascribe the observed effects to ALAN. Likewise, the experimental installation of streetlights in a natural setting allowed us to disentangle the effects of ALAN from other aspects of urbanization such as pollution, noise, and habitat alteration, which confound most studies. Considering the results of our previous lab studies and the research discussed above, we think that our results provide a clear hint that ALAN in naturally dark environments can disrupt physiological rhythms of fish. These findings can be relevant to policy-makers and conservation practitioners to improve the success of future management and conservation interventions (Cooke *et al.*, 2013).
